# Right internal jugular vein ectasia: unmasking a rare cause of neck swelling in an adult Nepalese male – a case report

**DOI:** 10.1097/MS9.0000000000003166

**Published:** 2025-03-19

**Authors:** Mandeep Kumar Yadav, Ashok Paudel

**Affiliations:** a Kathmandu Medical College, Sinamangal, Kathmandu, Nepal; b Nepal Bharat Maitri Hospital, Mitrapark, Kathmandu, Nepal

**Keywords:** case report, jugular vein ectasia, neck swelling, Nepal, venous anomaly

## Abstract

**Introduction:**

Internal jugular vein ectasia (IJVE) is a rare venous anomaly presenting as unilateral neck swelling that is predominantly observed in childhood and infrequently reported in adults. Notably, fewer than 100 cases have been documented worldwide, emphasizing its rarity.This case highlights a 61-year-old Nepalese male with IJVE, notable for its atypical adult onset and right sided presentation.

**Case presentation:**

The patient presented with painless, intermittent swelling that worsened with activities increasing intrathoracic pressure. Clinical examination revealed a soft, pulsatile, fusiform swelling in the right lower neck, well-demarcated along the sternocleidomastoid muscle. The swelling enlarged during the Valsalva maneuver, and ultrasound confirmed IJVE without complications. Given his history of stroke and residual dysphagia, it is important to note that the IJVE’s onset appears coincidental rather than causally related.

**Discussion:**

The exact cause of IJVE remains unclear. Often discovered incidentally, the diagnosis requires differentiation from other neck masses. A detailed history, physical examination, and early use of ultrasound – a low-cost, low risk, and highly accessible imaging modality with high availability and minimal financial burden worldwide – are critical for accurate diagnosis. Conservative management is typically sufficient, with surgery reserved for significant symptoms or cosmetic concerns.

**Conclusion:**

This case underscores the importance of considering IJVE in the differential diagnosis of neck masses, particularly in adults with atypical symptoms. Thorough evaluation and imaging – especially early ultrasound use – play a crucial role in accurate diagnosis and avoiding unnecessary interventions.

## Introduction

Internal jugular vein ectasia (IJVE) is a rare condition where the internal jugular vein becomes unusually dilated. It often appears as a soft, compressible swelling on one side of the neck, which becomes more noticeable during activities that increase intrathoracic pressure, such as straining, coughing, or sneezing. While it’s more commonly seen in children, it can also occur in adults, though cases in adults are rare with fewer than 50 cases reported globally and often go unnoticed because they are usually asymptomatic^[1,2]^.

The etiology of internal jugular vein ectasia remains unclear and controversial due to the limited number of reported cases in the neck. Epidemiologically, IJVE is extremely rare, with fewer than 100 cases reported in the literature, thereby necessitating a high index of suspicion. Although more common in children, adult presentations, such as in the case described here, are infrequent and often discovered incidentally.

**HIGHLIGHTS**

- Internal jugular vein ectasia (IJVE) in a 61-year-old Nepalese male, a condition more commonly found in children.
- The patient presented with painless, intermittent right neck swelling, exacerbated by activities such as the Valsalva maneuver, along with a history of stroke.
- Ultrasound identified fusiform dilatation of the right internal jugular vein with normal venous flow, highlighting the importance of non-invasive imaging in diagnosing IJVE.
- Owing to the absence of symptoms and cosmetic concerns, the patient opted for conservative management with regular follow-up.
- This case highlights the need to include IJVE in the differential diagnosis of neck masse in adults, particularly when swelling occurs during straining, and advocates for conservative treatment when appropriate.

Here, we present the case of a 61-year-old Nepalese male with unilateral neck swelling, highlighting the role of ultrasound in diagnosing internal jugular vein ectasia. This case report has been prepared in accordance with the SCARE guidelines[3].

## Case presentation

A 61-year-old Nepalese man visited our out-patient clinic with a painless, intermittent swelling on the right side of his neck that he had noticed about three years ago. The swelling was insidious at onset, and gradually progressive, with prominence on leaning forward or straining. He denies any associations of voice changes, dyspnea, or dysphagia, chest pain, or unwanted weight loss. His medical history was significant for cerebrovascular accidents three years back, resulting in weakness in the left upper limb, facial paralysis on the right side, dysarthria, and dysphagia. Besides, he had been hypertensive for the past four years and on medication with amlodipine 5 mg and losartan 50 mg per day.

**Figure 1.**
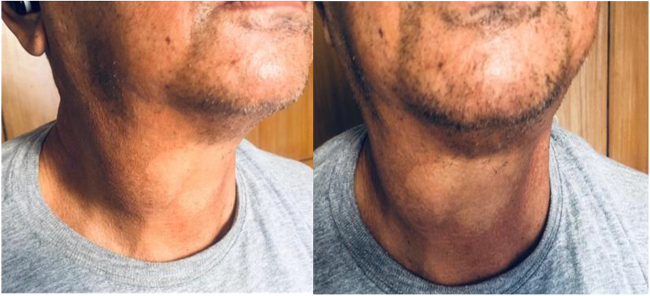
Patient with golf ball-sized swelling located on the right lower neck. The swelling is highlighted with an arrow.

Physical examination revealed a soft, pulsatile, fusiform swelling in the lower right neck. The swelling was anteromedially related to the sternocleidomastoid muscle, and approximately the size of a golf ball. It was well-defined without overlying erythema or skin changes. The swelling increased in size with the Valsalva maneuver (Fig. 1) and was non-tender but compressible on palpation. Auscultation revealed no bruits. A detailed neurological examination showed motor aphasia, a cranial nerve VII deficit, and right-sided limb weakness. The remainder of the physical examination was unremarkable.

**Figure 2.**
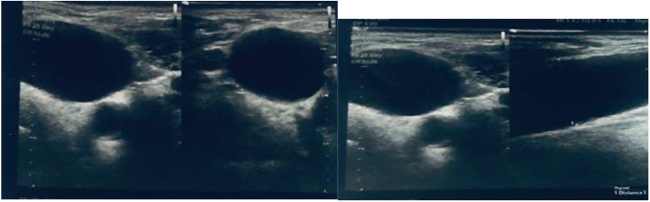
Ultrasound image of the mass showing fusiform dilatation of the right internal jugular vein, measuring 2.2 cm at its widest transverse diameter, which exceeds the normal range.

Ultrasound of the neck revealed a fusiform dilatation of the right internal jugular vein, measuring 2.2 cm at its widest transverse diameter at the level of the thyroid gland (Fig. 2). Doppler interrogation demonstrated normal venous spectral waveforms and flow, with the vein being fully compressible. The contralateral internal jugular vein was unremarkable, measuring 1.0 cm in diameter at the same level.

Given the patient’s history of stroke, a magnetic resonance imaging (MRI) of the brain was performed which revealed linear areas of cystic encephalomalacia with gliosis in the bilateral frontal lobes and hemosiderin staining peripherally on the left side consistent with prior infarctions, without evidence of acute ischemic changes. It is important to highlight that while the patient’s residual stroke symptoms (including dysphagia) are noted, no causal relationship with the development of IJVE is implied; the two conditions are considered coincidental.

The condition was managed conservatively, with precautions discussed at each visit, including maintaining proper hygiene to reduce the risk of infection and avoiding activities that could increase the likelihood of trauma or bleeding. During follow-up assessments at 3 and 6 months, the lesion’s size remained stable, and no significant symptoms or complications were observed. The patient’s condition has been stable, and he is scheduled for annual follow-up visits to monitor the progression of the swelling.

## Discussion

IJVE is a rare vascular anomaly, typically unilateral, and often diagnosed in childhood.[4] Adult cases are rare and often incidentally discovered during imaging for unrelated conditions

The exact cause of IJVE remains unclear, and the limited epidemiological data complicate efforts to estimate its true prevalence.[5] In this case report, we presented a Nepalese adult male with neck swelling that worsened with leaning forward or straining. To our knowledge, this is the first documented case of adult-onset IJVE in a male from Nepal.

IJVE is more commonly observed in males, with a male-to-female ratio ranging from 2:1 to 3:2[6]. It is typically associated with neck swelling noticeable from birth[7] and is more frequently observed on the left side in adults, whereas in children, it tends to occur on the right side^[4,6,8]^. In our case, the ectasia was located on the right side of the neck, which mirrors the pediatric pattern and the condition was first noted three years prior to the patient’s presentation which makes it an interesting and atypical presentation.

Typically, IJVE manifests as a benign, asymptomatic swelling on the lateral side of the neck, which becomes more prominent with physical exertion, such as during straining or the Valsalva maneuver[9]. In our patient, the swelling was visible on the right side of the neck and did not cause significant morbidity, consistent with the usual benign course of the condition. Although some cases report mild symptoms such as discomfort during swallowing, hoarseness, or a sensation of a foreign body in the neck, as well as more severe manifestations like neck pain, Horner syndrome, or thrombosis^[9,10]^, our patient did not experience any of these symptoms, further confirming the benign nature of the condition in this case.

*Our patient has a history of stroke and residual symptoms, including dysphagia, but there is no evidence to suggest that these factors are related to the development of adult-onset IJVE. The occurrence of IJVE appears to be incidental and not directly linked to the patient’s prior stroke.*

The differential diagnosis for neck masses is broad. If intermittent localized swelling occurs exclusively during episodes of increased intrathoracic pressure – such as during straining, crying, or the Valsalva maneuver – possible diagnoses include laryngocele, inflation of pulmonary apical bullae, superior mediastinal mass, and other ectasias, with laryngocele being the most common[11]. Thus for an accurate diagnosis of IJVE, it is crucial to distinguish it from these conditions through thorough examinations and investigations. A Laryngoscopy can rule out a laryngocele, while thoracic computed tomography (CT) is necessary to exclude mediastinal cysts or tumors. However, in our case, an extensive workup of potential differentials was not performed since the patient had limited symptoms and examination findings. This is the limitation of our study.

In order to diagnose IJVE various imaging techniques including venography, CT, MRI, and color flow Doppler can be used.However, ultrasonography is often sufficient and preferred worldwide due to its non-invasive nature, avoidance of radiation, high availability, and minimal financial burden^[8,12,13]^. Ultrasound effectively identifies venous structures and typically shows a flattened spectral waveform, making it the preferred tool for diagnosing IJVE[8]. Moreover, ultrasound is easily available and minimally burdensome financially. Our case was also diagnosed using the same and hence highlights the justification for using ultrasound imaging in the early workup of such presentations.

The management for typical benign and asymptomatic cases of IJVE without cosmetic concerns is conservative. The conservative approach involves annual clinical and ultrasound evaluations with regular counselling to monitor for any changes in the lesion and to take protective measures to minimize the risk of hemorrhage and infection[14]. Surgery may be considered in selected cases owing to risks such as rupture, intramural thrombosis leading to pulmonary embolism, or for cosmetic and psychological concerns. The preferred surgical method is constriction suture venoplasty with encapsulation, although ligation or resection of the affected vein may be necessary, particularly if the internal jugular vein is involved[12]. Our patient was managed conservatively with regular follow-ups.

In summary, Jugular vein ectasia is often misdiagnosed or improperly managed due to its rarity and asymptomatic presentation. Thus, when a patient presents with intermittently increasing neck swelling or masses, early use of ultrasound with the Valsalva maneuver should be considered to ensure accurate diagnosis and appropriate management.

## Conclusion

In conclusion, IJVE is a rare but important differential diagnosis of unilateral neck swelling in adults. This case highlights the importance of thorough evaluation and consideration of less common causes of neck swellings, such as jugular vein ectasia, particularly when the swelling is soft, compressible, and increases with activities that increase intrathoracic pressure. While the condition is generally benign, accurate diagnosis is crucial to prevent unnecessary interventions. Conservative management with regular follow-up is typically sufficient in asymptomatic cases, whereas surgical intervention may be reserved for patients with significant symptoms such as persistent pain, recurrent thrombophlebitis, and compression of adjacent structures leading to discomfort or dysfunction, or severe cosmetic deformity affecting the quality of life. Patients should be reassured of the condition’s benign nature and advised that extensive diagnostic procedures are unnecessary and regular follow-up is sufficient for asymptomatic cases without cosmetic concerns.

## Data Availability

All data are provided within this review and data from the original published papers noted in this review.

## Author agreement statement

We confirm that this manuscript is original, has not been previously published before, and is not currently being considered for publication elsewhere. Furthermore, we confirm that all the authors have approved the manuscript and that there are no other persons who satisfied the criteria for authorship and are not listed above. The orders of the authors listed in the manuscript have been approved by all of the authors.
